# An RNA Interference Screen for Genes Required to Shape the Anteroposterior Compartment Boundary in *Drosophila* Identifies the Eph Receptor

**DOI:** 10.1371/journal.pone.0114340

**Published:** 2014-12-04

**Authors:** Daiki Umetsu, Sebastian Dunst, Christian Dahmann

**Affiliations:** 1 Institute of Genetics, Technische Universität Dresden, Dresden, Germany; 2 Max Planck Institute of Molecular Cell Biology and Genetics, Dresden, Germany; MRC, University College of London, United Kingdom

## Abstract

The formation of straight compartment boundaries separating groups of cells with distinct fates and functions is an evolutionarily conserved strategy during animal development. The physical mechanisms that shape compartment boundaries have recently been further elucidated, however, the molecular mechanisms that underlie compartment boundary formation and maintenance remain poorly understood. Here, we report on the outcome of an RNA interference screen aimed at identifying novel genes involved in maintaining the straight shape of the anteroposterior compartment boundary in *Drosophila* wing imaginal discs. Out of screening 3114 transgenic RNA interference lines targeting a total of 2863 genes, we identified a single novel candidate that interfered with the formation of a straight anteroposterior compartment boundary. Interestingly, the targeted gene encodes for the Eph receptor tyrosine kinase, an evolutionarily conserved family of signal transducers that has previously been shown to be important for maintaining straight compartment boundaries in vertebrate embryos. Our results identify a hitherto unknown role of the Eph receptor tyrosine kinase in *Drosophila* and suggest that Eph receptors have important functions in shaping compartment boundaries in both vertebrate and insect development.

## Introduction

The precise organization of cells within tissues is important for animal development. The establishment of intricate tissue patterns often requires the separation of cells with distinct fates and functions into specific regional domains [1]. These patterns can initially be imprecise and can be sharpened during tissue development. However, cell proliferation and cell movements can lead to cell intermingling and thereby challenge precise tissue organization. Although the precise spatial organization of cells within tissues is important for animal development and homeostasis, the molecular mechanisms underlying it are not well understood.

The formation of straight boundaries between regional domains within tissues termed compartments is a well-known example of tissue organization [2], [3], [4], [5], [6], [7], [8], [9], [10], [11]. Compartment boundaries are lineage restrictions that separate cells with distinct identities. Signals across compartment boundaries induce organizing centers along compartment boundaries that specify growth and patterning within the tissue [12]. Compartment boundaries play thus a pivotal role during tissue development. Several compartment boundaries have been identified, including the mid-hindbrain boundary and rhombomere boundaries of vertebrate embryos [13], [14], and the anteroposterior (AP) and dorsoventral (DV) compartment boundaries of larval wing imaginal discs of *Drosophila* [15], [16].

A common mechanism to establish and maintain compartment boundaries is the exchange of signals across the boundary [12]. In the vertebrate hindbrain, signaling mediated by Eph receptor tyrosine kinases and their ephrin ligands is required for separating cells from neighboring rhombomeres [17], [18], [19], [20]. Ephrin-Eph signaling has been proposed to mediate repulsion as well as de-adhesion between cells from neighboring compartments [21], [22]. In the *Drosophila* wing imaginal disc, maintenance of the AP boundary requires signaling by Hedgehog and Decapentaplegic (Dpp, a BMP2/4 ortholog) and the activities of the *engrailed* and *invected* selector genes in posterior cells [23], [24], [25], [26], [27]. The selector gene *apterous* and Notch signaling are required to maintain the DV boundary [28], [29], [30]. Recent data indicate that local increases in mechanical tension at adherens junctions play an important role in maintaining the straight morphology of both the AP and DV boundaries [31], [32]. How the signaling and selector gene activities, however, alter mechanical tension to maintain straight compartment boundaries remains poorly understood.

Here, to identify additional genes required to maintain the straight shape of the AP boundary of *Drosophila* wing imaginal discs, we first developed an assay that allows to reliably and quickly test for cell sorting defects along the AP boundary in larval wing imaginal discs. We then combined this assay with RNA interference to test 3114 transgenic RNAi lines for their influence on the shape of the AP boundary. We identified one candidate gene, *Eph*, which encodes for the single *Drosophila* Eph receptor tyrosine kinase.

## Results

### An assay to identify genes required to maintain the shape of the AP boundary

**Figure 1:**
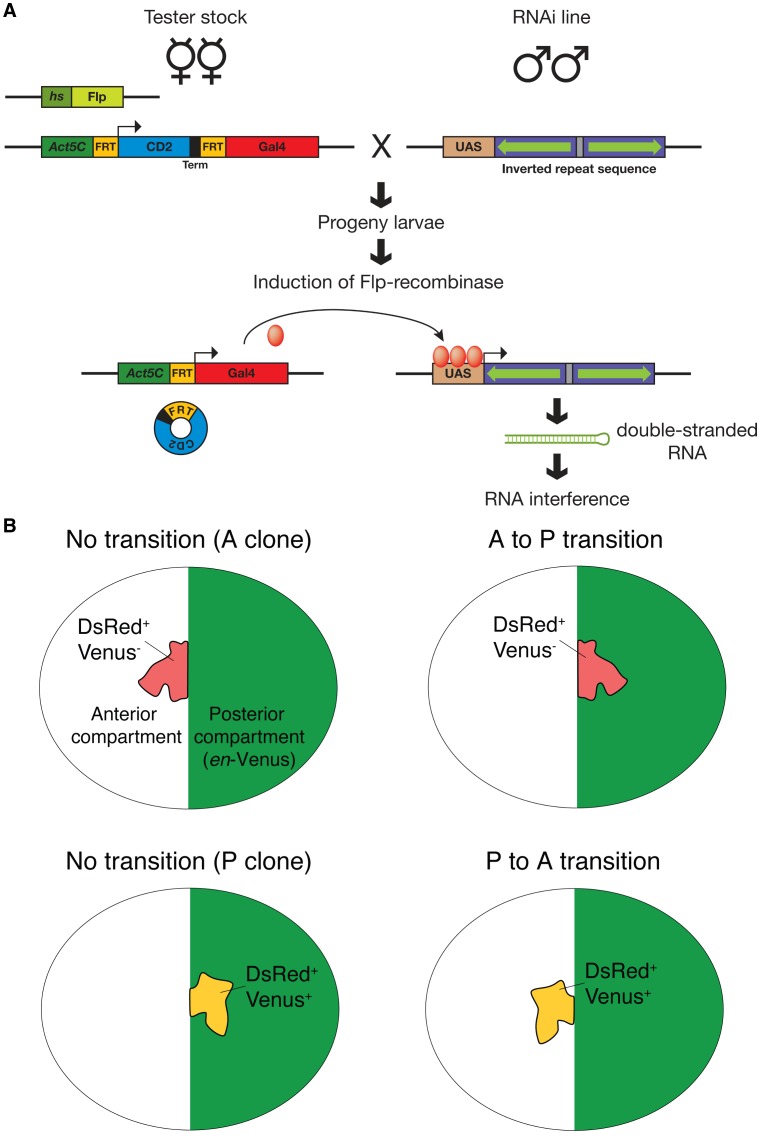
An assay to identify genes required to maintain the straight shape of the AP boundary. A. Scheme of tester stock and cross to generate clones of cell expressing double-stranded RNA. The tester stock contains a *Flp* recombinase transgene under control of the heat-shock inducible promoter hsp70 (*hs*) on chromosome I [36] and the *engrailed-Venus* transgene on chromosome II (not shown). Chromosome III contains a transgene composed of the *Act5C* promoter followed by a FRT site, the coding sequence for the mouse transmembrane protein CD2 followed by a transcriptional terminator (Term), a second FRT site, and finally Gal4. We have recombined on the same chromosome a transgene expressing DsRed under control of UAS sequences (not shown). The tester stock is crossed to individual RNAi fly lines. The resulting progeny larvae are heat-shocked to express the Flp recombinase that in turn induces recombination between the two FRT sites on the tester stock. This recombination places Gal4 under the direct control of the *Act5C* promoter leading to Gal4 expression specifically in the clones of cells. Gal4 then binds to the UAS sequences in the RNAi line and directs expression of double-stranded RNA that leads to RNA interference. At the same time, Gal4 will induce expression of DsRed (not shown) in the same cells. B. Scheme of the assay. The central part of the wing imaginal disc is schematically drawn as an oval. A Venus fluorescent protein (green) is specifically expressed in all cells of the posterior compartment under control of the promoter sequences of the *engrailed* gene (*en*-Venus). Clones of cells expressing double stranded RNA are identified by co-expression of DsRed (red). Examples on the left depict wild-type clones located in the anterior (A) or posterior (P) compartments. Examples on the right depict a anterior clone mis-segregating into the posterior territory (top) and an posterior clone mis-segregating into the anterior territory (bottom) of the wing disc. Clones co-expressing DsRed and Venus are depicted in yellow.

We sought to identify genes required to maintain the shape of the AP boundary by RNA interference. Expression of double-stranded hairpin RNA from a transgene can result in the RNA interference mediated knock-down of gene function in *Drosophila* [33]. We developed an assay to assess whether the knock-down of a particular gene influences the shape of the AP boundary. In this assay, double-stranded hairpin RNA is expressed in clones of cells using the Gal4-UAS [34] and FRT-flp [35], [36] systems (Fig. 1A). Clones of cells located along the AP boundary were tested whether or not they influenced the shape of the AP boundary (Fig. 1B). To identify the clones of cells and the AP boundary, we used two fluorescent reporters. One reporter (DsRed) identified the clone of cells that expressed the double-stranded RNA and the second reporter (*en*-Venus) labeled all cells of the P compartment and thus visualized the AP boundary (see Fig. 1B). Hand-dissected wing imaginal discs were then viewed under a fluorescence microscope. Segregation defects were recognized as *en*-Venus negative clones of cells located in the posterior territory of the wing imaginal disc or by *en*-Venus positive clones in the anterior territory of the wing imaginal disc (Fig. 1B).

**Figure 2:**
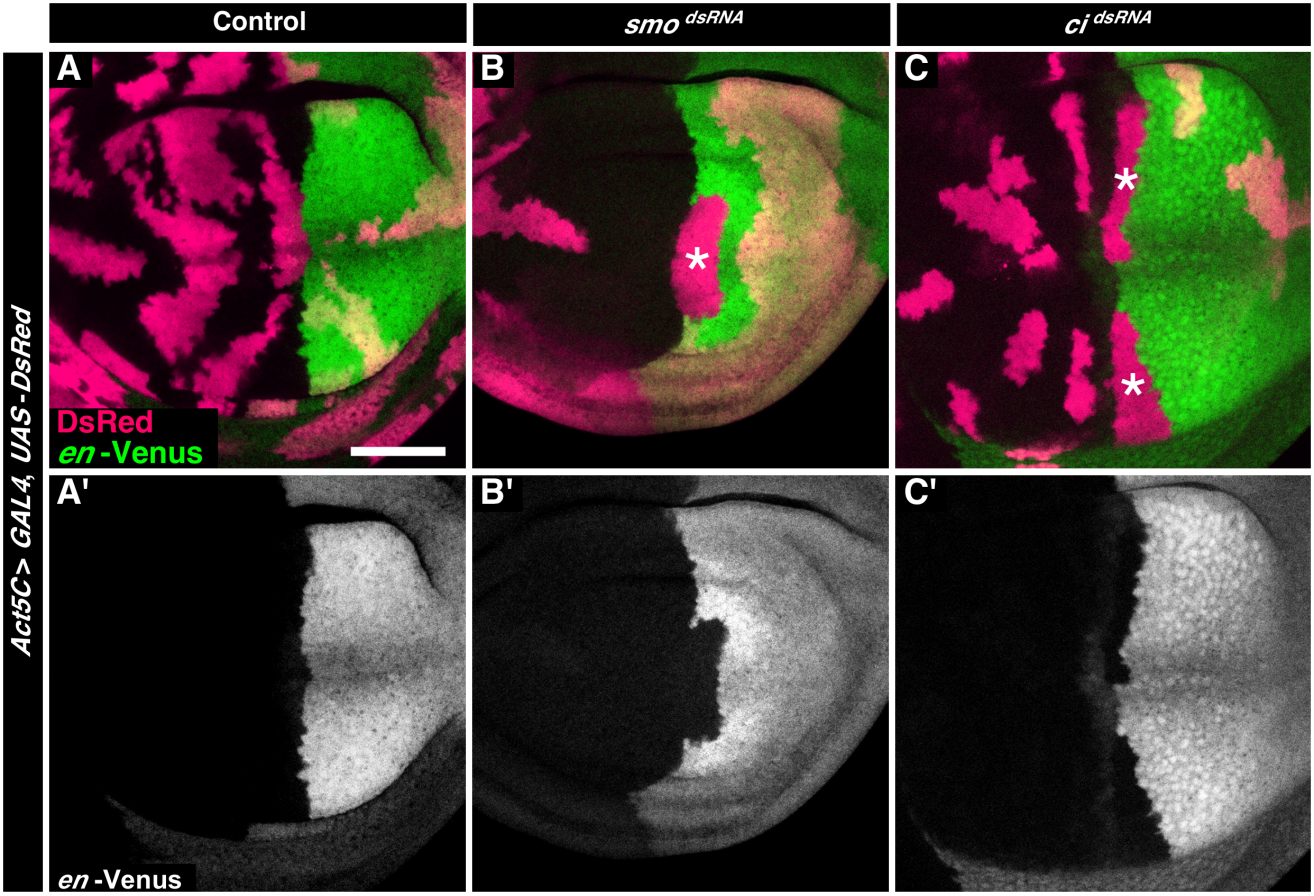
Validation of assay. **A–C.** Wing discs expressing (A) no double-stranded RNA (control), (B) double-stranded RNA targeting *smo*, and (C) double-stranded RNA targeting *ci* in clones of cells marked by the expression of DsRed (red). Cells of the posterior compartment are labeled by expression of Venus under control of the *engrailed* gene (*en*-Venus, green). In (B) and (C), anterior clones along the AP boundary mis-segregate into the posterior territory of the wing disc (asterisks). Scale bar is 50 µm.

To validate the assay we used RNAi lines targeting the genes *smoothened* and *ci*, two genes coding for a Hedgehog signal transducer and a transcription factor, respectively, that are known to be required in A cells to maintain the straight shape of the AP boundary [23], [24], [26]. Control clones of cells did not alter the shape of the AP boundary (Fig. 2A). By contrast, anterior clones of cells expressing double-stranded RNA targeting *smo* or *ci* altered the straight shape of the AP boundary and took up positions normally only occupied by posterior cells (Fig. 2B, C). This clonal behavior was reminiscent of clones of cells mutant for *smo* or *ci* in typical mosaic experiments using antibody stainings [23], [24], [26], indicating that these RNAi lines and the assay work faithfully.

### RNA interference screen

**Figure 3:**
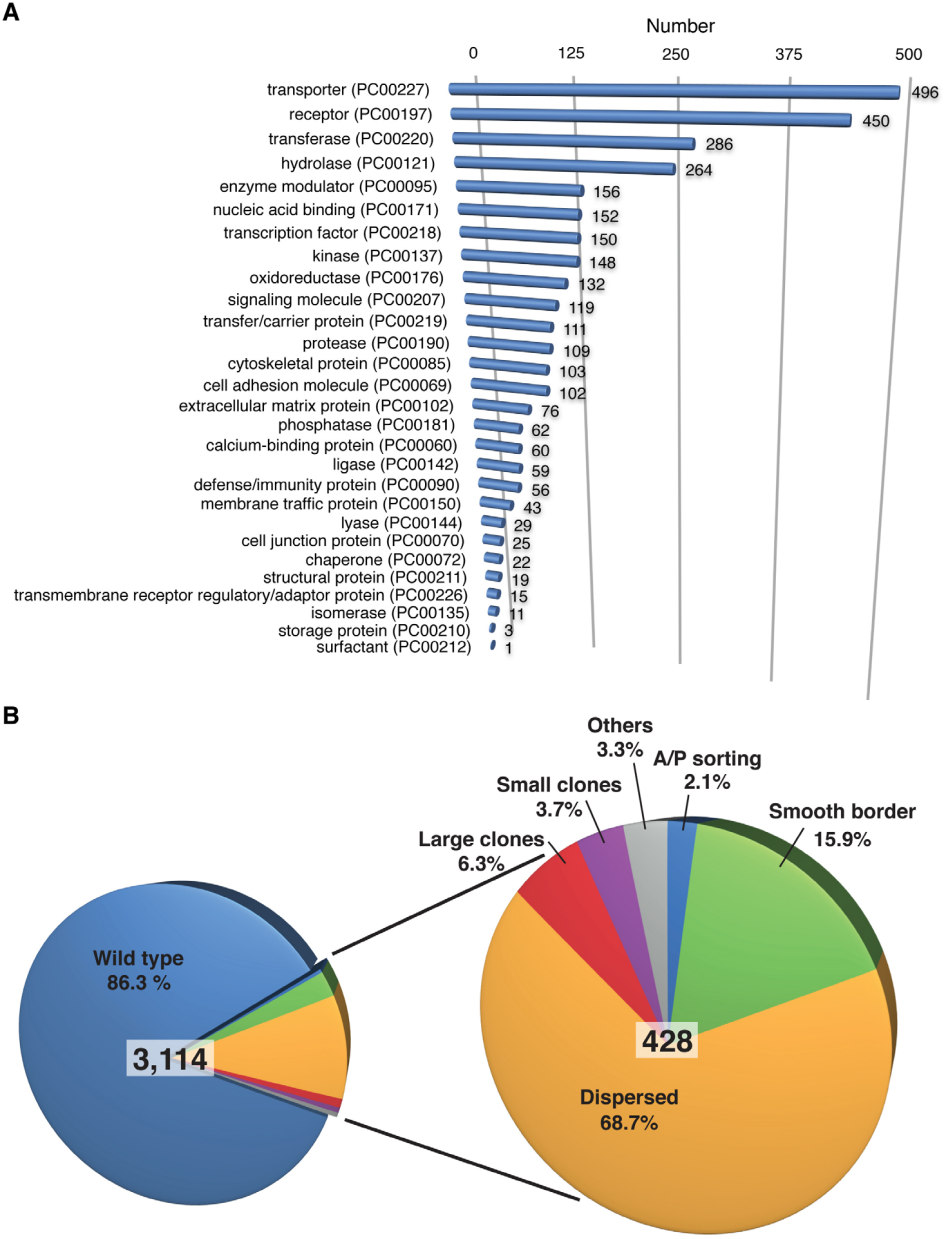
Summary of RNA interference screen. **A.** Number of RNAi lines screened categorized for the class of targeted gene products. **B.** Number of the analyzed RNAi lines and fractions of the phenotypic categories as a percentage.

We next used the assay to screen 3114 selected RNAi lines targeting a total of 2863 genes [37]. We mainly sought to analyze RNAi lines that targeted genes encoding transmembrane proteins. Transmembrane proteins can mediate cell-cell communication, cell-cell recognition or cell adhesion that might be important for maintaining the shape of the AP boundary. 3181 genes encoding transmembrane proteins were identified in *Drosophila* based on sequence analysis (Krystyna Keleman, personal communication). We analyzed RNAi lines targeting 2204 of these genes encoding for transmembrane proteins. In addition, we analyzed RNAi lines targeting genes involved in cytoskeletal (114 genes, 4.0%) or transcriptional (127 genes, 4.4%) regulation. A classification of the proteins encoded by the targeted genes based on protein function [38] is detailed in Fig. 3A. The persons performing and analyzing the RNA interference screen had no knowledge of the identity of the targeted gene products.

**Figure 4:**
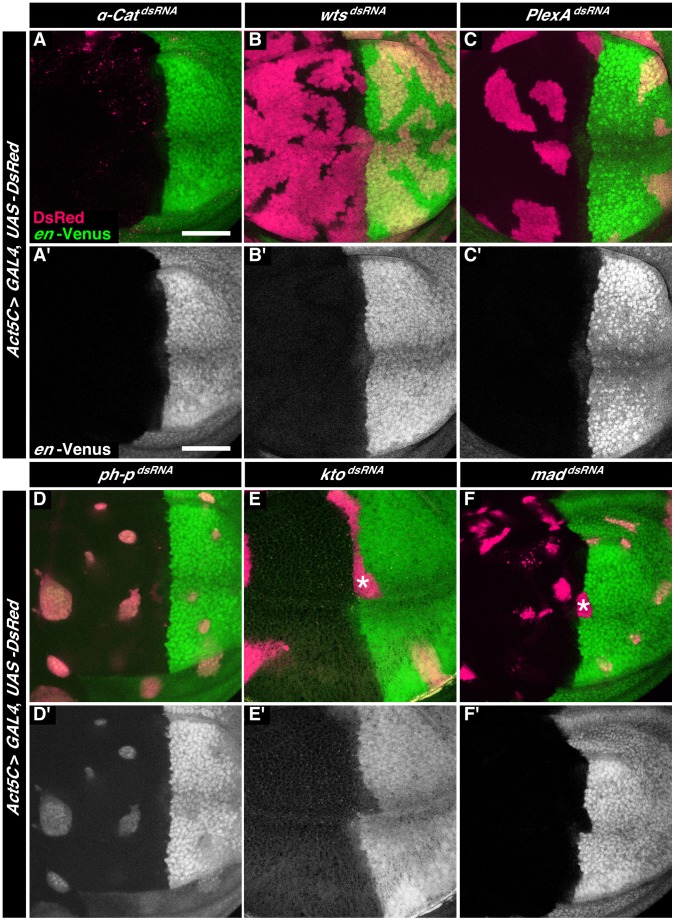
Examples of phenotypes observed in the RNA interference screen. **A–F.** Wing imaginal discs displaying clones of cells expressing double-stranded RNA targeting (A) *α-cat*, (B) *wts*, (C) *PlexA*, (D) *ph-p*, (E) *kto*, and (F) *mad*. The clones of cells are identified by co-expression of DsRed (red); cells of the posterior compartment are labeled by expression of Venus under control of the *engrailed* gene (*en*-Venus, green). Clones of cells distorting the AP boundary are marked by an asterisk. Scale bars are 50 µm.

428 out of the 3114 RNAi lines resulted in clones of cells that in terms of size, shape, appearance or sorting at the AP boundary differed from control clones (Fig. 3B, Table S1). 68.7% of these 428 RNAi lines led to clones of cells that did not have a coherent appearance as control clones had, but in which cells were rather dispersed. An example for such an RNAi line is line 19182 (VDRC) (Fig. 4A), which targets the gene *α-catenin*. *α-catenin* encodes a component of adherens junctions that is essential for cell survival in wing imaginal discs [39]. We speculate that the dispersed clone appearance is due to cell death. 3.7% and 6.3% of the RNAi lines yielded clones that were smaller or larger than control clones, respectively. For example, RNAi line 9928 (VDRC), which targets the gene *warts*, resulted in larger clones of cells (Fig. 4B). These data are consistent with the known function of Warts as a tumor suppressor [40]. These differences in clone size are thus consistent with a role of the targeted genes in growth control. 15.9% of the 428 RNAi lines gave rise to clones that had a smooth clonal border, indicating that the targeted genes play a role in cell-cell recognition or cell-cell adhesion. Clones expressing these double-stranded RNAs, however, did not influence the shape of the AP boundary. An example of such an RNAi line is line 27240 (VDRC) (Fig. 4C), which targets the gene *Plexin A*. *Plexin A* encodes a transmembrane receptor protein tyrosine kinase required, for example, for axon guidance in the nervous system [41]. A further RNAi line that fell into this class was 10679 (VDRC), which targets *ph-p*, a member of the Polycomb group of genes. In contrast to the other lines in this category, expression of double-stranded RNA from this line resulted in the expression of the Venus marker in anterior cells (Fig. 4D), consistent with the function of Polycomb group genes to repress homeotic genes. Finally, nine out of the 428 RNAi lines (2.1%) resulted in clones of cells that did display sorting defects along the AP boundary and locally disturbed the shape of the AP boundary. Six of these RNAi lines targeted genes encoding components of signal transduction pathways or transcriptional regulators that were previously shown to be required to maintain the A/P boundary (*ci*, *smo*, *punt*, *mad*, *kto* (2x) [23], [24], [26], [27], [42]; Fig. 4E–F, Table S1). The ‘blind’ identification of these 6 RNAi lines furthermore confirms the feasibility of our screen.

The remaining three of the nine RNAi lines were 11796 (VDRC), 27465 (VDRC), and 6481 (VDRC), targeting the genes *amos*, *Sce*, and *CG32442*, respectively. RNA interference is prone to sequence-specific off-target effects [33], [43]. We therefore tested whether the distortion of the AP boundary was due to the targeting of *amos*, *Sce*, and *CG32442*, or rather some unrelated gene. Clonal analysis using the mutant alleles *amos^1^*, or *Sce^1^* did not reveal cell sorting defects at the AP boundary (data not shown). Moreover, using a second RNAi line targeting *CG32442* (101469, VDRC) we failed to detect the sorting defects that we had seen with the RNAi line 6481 targeting *CG32442* (Table S1). Therefore, we could not establish a role for *amos*, *Sce* or *CG32442* in cell sorting at the AP boundary.

### Identification of the Eph receptor as a candidate of the RNAi screen

**Figure 5:**
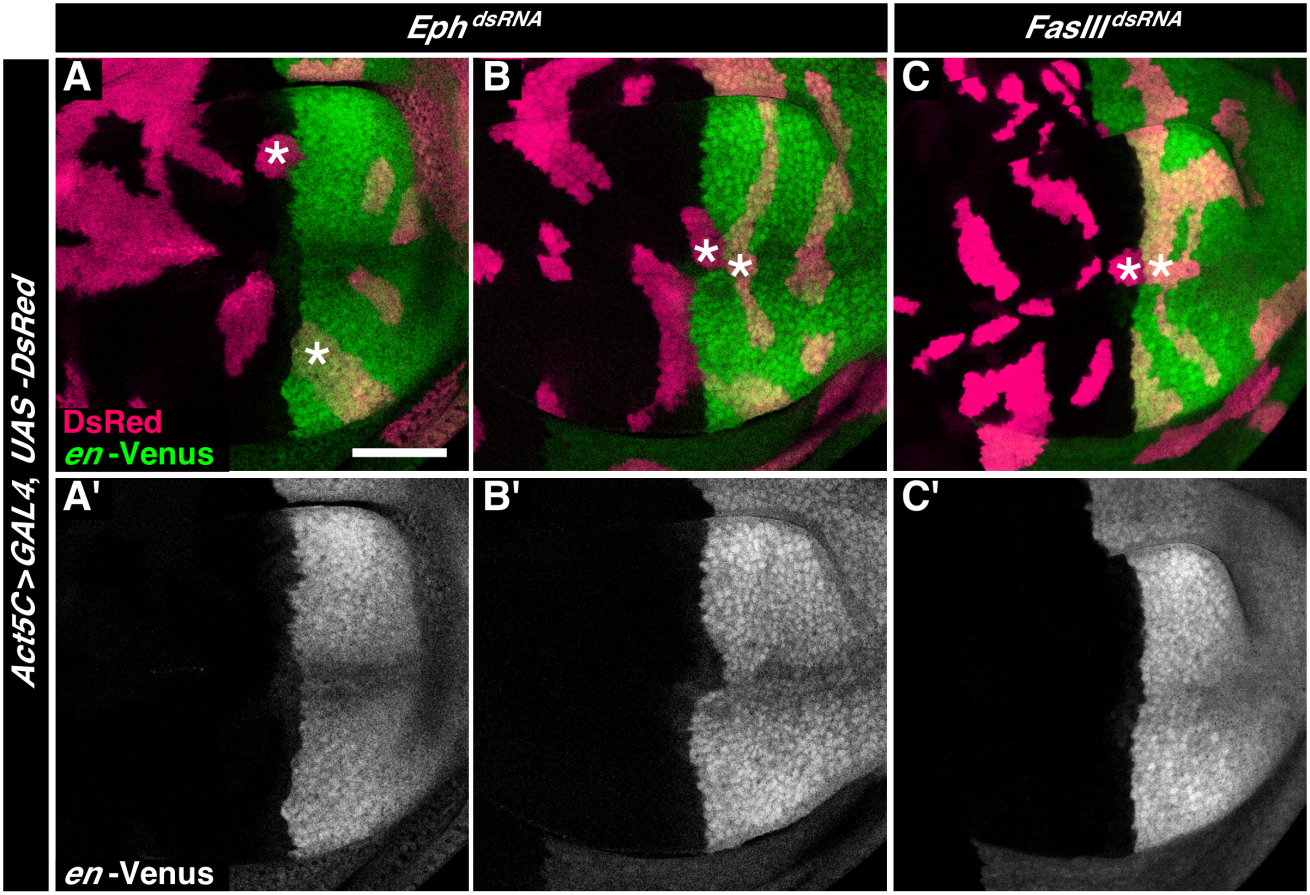
Clones of cells expressing double-stranded RNA targeting the *Eph* gene distort the AP boundary. **A–C.** Wing imaginal discs displaying clones of cells expressing double-stranded RNA targeting (A–B) *Eph* or (C) *FasIII.* The clones of cells are identified by co-expression of DsRed (red); cells of the posterior compartment are labeled by expression of Venus under control of the *engrailed* gene (*en-*Venus, green). (A) Individual clones abutting the AP boundary from either the A or P compartment do not influence the shape of the boundary (asterisk). (B) Two clones expressing *Eph^dsRNA^* (ID-Number 4771 (VDRC)) located in different compartments sharing a common interface along the AP boundary locally distort the shape of the AP boundary (asterisks). (C) Two clones expressing *FasIII^dsRNA^* located in different compartments sharing a common interface along the AP boundary do not distort the shape of the AP boundary (asterisks). Scale bar is 50 µm.

The roundish shape of clones is a signature of cell sorting [44]. We therefore re-screened RNAi lines that in the primary screen displayed a rounded shape for causing defects in the shape of the A/P boundary. In no case did we find that individual clones of cells located along the AP boundary influenced boundary shape (e.g. Fig. 5A, and Table S1). Interestingly, however, we found that expression of three RNAi lines, 43454, 10064, and 6545, targeting the *CG10176*, *CG9416* and *Eph* genes, respectively, resulted in the following phenotype: when two clones located in different compartments shared a common interface along the AP boundary, then the shape of the AP boundary was locally distorted (Fig. 5B and data not shown). Abutting clones of cells from different compartments displaying smooth clone borders as a result of expression of double-stranded RNA targeting *PlexA* (n = 10 clones), *ds* (n = 21 clones), or *FasIII* (n = 6 clones) did not influence the shape of the AP boundary (Fig. 5C, data not shown). This suggests that two abutting clones with smooth clone borders do not *per se* disturb the shape of the AP boundary.

We next tested whether the distortion of the AP boundary was due to the knock-down of the two genes *CG10176* and *CG9416* by using additional RNAi lines. Expression of the additional RNAi line targeting *CG10176* (104538, VDRC) or targeting *CG9416* (106330, VDRC) resulted in clones that had a rough border, similar to control clones, and did not result in sorting defects at the AP boundary (Table S1). Thus, roles for the genes *CG10176* or *CG9416* in cell sorting at the AP boundary could not be established.

**Figure 6:**
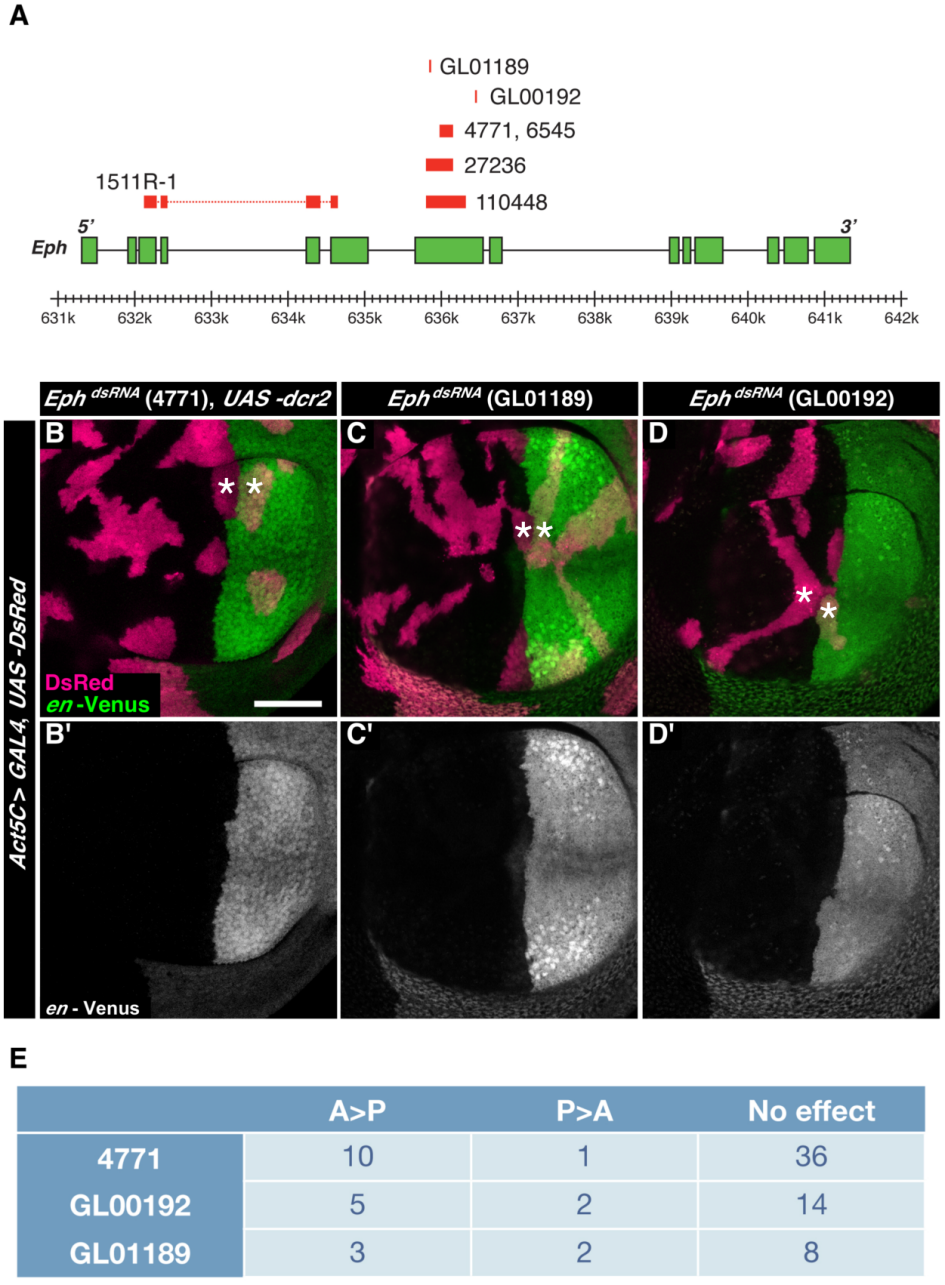
Validation of the phenotype of *Eph* RNAi lines. **A.** Exon-intron structure of the *Eph* gene and regions targeted by the indicated RNAi lines. **B–D.** Wing imaginal discs displaying clones of cells expressing double-stranded RNA targeting *Eph* using the RNAi lines (B) 4771, (C) GL01189 or (D) GL00192. The clones of cells are identified by co-expression of DsRed (red); cells of the posterior compartment are labeled by expression of Venus under control of the *engrailed* gene (*en-*Venu*s*, green). Two clones expressing *Eph^dsRNA^* located in different compartments sharing a common interface along the AP boundary locally distort the shape of the AP boundary (asterisks). In (B), *dcr2* is co-expressed to increase the efficiency of RNAi. **E.** Number of cases where two clones expressing double-stranded RNA targeting *Eph* located in different compartments sharing a common interface along the AP boundary distort the AP boundary towards the posterior side (A>P), towards the anterior side (P>A), or do not distort the AP boundary (no effect). Scale bar is 50 µm.

Finally, we used six additional RNAi lines to test whether the distortion of the AP boundary was due to the targeting of *Eph*. The regions of the Eph gene targeted by these RNAi lines are shown in Fig. 6A. RNAi lines 1511R-1, GL01189, GL00192, and 4771 were targeting non-overlapping regions of the *Eph* gene (Fig. 6A). Clones expressing double-stranded RNAs from any of these four additional RNAi lines had smooth edges (Fig. 6B–D and data not shown). Moreover, clones interfacing from the anterior and posterior side did disturb the shape of the AP boundary, similar to the RNAi line originally used in the screen (Fig. 6B–D and data not shown). In the case of clones targeting *Eph*, the AP boundary could either be distorted towards the anterior or towards the posterior side, although distortions to the posterior side were more frequently observed (Fig. 6B–E). Clones targeting *Eph* did not distort the shape of the DV boundary (Fig. S1A, n = 15 pairs of clones interfacing from the dorsal and ventral side). These data indicate a specific role of the Eph receptor in shaping the AP boundary.

## Discussion

We have performed an RNA interference screen to identify genes required to shape the AP boundary in wing imaginal discs. The screen is based on a mosaic assay that uses fluorescent proteins to identify at the same time clones of cells expressing double-stranded RNA and the AP boundary of wing imaginal discs. We have screened RNAi lines mainly targeting genes encoding transmembrane proteins and have identified RNAi lines that show defects in several processes, including growth, survival, and sorting at the AP boundary. In particular, we have identified the *Eph* gene as a novel candidate required to shape the AP boundary.

### A fluorescent protein-based assay

Previous attempts to identify novel genes involved in maintaining the shape of the AP boundary in wing imaginal discs have relied on defects in the veination pattern of the adult wing as a readout of sorting defects during wing development [45]. Defects in the veination pattern are, for example, observed when clones of cells deficient for Hedgehog signal transduction ‘cross’ from the anterior to the posterior territory of the developing wing [45]. However, for example cells mutant for *thickveins*, a receptor of the BMP2/4 homolog Dpp, show segregation defects at the AP boundary at larval stages [27], but these cells can then be extruded from the tissue resulting in a wild-type veination pattern in the adult wing [46], [47]. To circumvent problems associated with analyzing veination pattern in the adult as indication of cell sorting defects, we developed a mosaic assay that allows to reliably and quickly test for cell sorting defects along the AP boundary in wing imaginal discs during larval stage. Since the assay is based on fluorescent proteins, time-consuming immunostainings, as they usually done in mosaic analysis [48], are avoided. This allows the assay to be performed rapidly and at high throughput.

### RNAi screen

RNAi screens, both cell-based and *in* *vivo*, have been recently used to identify genes in various processes, including growth, signal transduction, cancer, cell differentiation, and aging. [33], [43]. One advantage of RNAi screens over mutagenesis screens is that the observed phenotype can be readily linked to the gene targeted by the RNAi line. Moreover, since each gene is targeted only by one or a few RNAi lines, the number of animals that needs to be screened is dramatically reduced compared to mutagenesis screens.

We have combined our mosaic assay with RNAi to identify genes important for maintaining the normal shape of the AP boundary in wing imaginal discs. Our screen has identified genes that were previously known to be important for maintaining a straight AP boundary, thus validating the screen. Some genes known in this process, however, including the selector gene *engrailed* [25], did not score positive in our screen (Table S1), thus showing also limitations of the approach. The screen has also uncovered a number of novel candidates in processes like growth or cell survival. Further analyses of these candidates may yield novel insights into these processes.

The screen has identified a single novel candidate gene, *Eph*, which is required for maintaining the normal shape of the AP boundary. Several reasons could account for this limited number of candidates. First, we have only screened RNAi lines targeting 2863 of the estimated 13.600 genes encoded in the *Drosophila* genome [49]. Second, a number of RNAi line may not have significantly knocked-down the targeted genes. Dietzl et al. indeed estimate that only 60% of the VDRC RNAi lines efficiently reduce target gene activity [37]. Third, genes required to maintain the AP boundary may act redundantly and are thus difficult to uncover in genetic screens perturbing the function of one gene at a time. The use of a sensitized genetic background or the co-expression of multiple RNAi lines may help to overcome genetic redundancy. Fourth, genes required to maintain the shape of the AP boundary may have, in addition, essential cellular functions and may thus result in cell lethality when knocked-down. Consistent with this possibility, a large fraction of RNAi lines led to a ‘dispersed’ phenotype that likely reflects cell death. Since the clones of cells expressing the double-stranded RNA lacked a coherent appearance they could not be assessed for cell sorting defects at the AP boundary. Co-expression of the apoptosis inhibitor p35 [50] with these RNAi lines resulted in the extrusion of the clonal cells (data not shown), again precluding the assessment of the sorting behavior of these cells at the AP boundary.

### *Eph* candidate gene

We have identified in our screen the *Eph* gene as a candidate required to maintain a straight boundary between anterior and posterior compartments in *Drosophila* wing imaginal discs. The Eph receptor belongs to a large family of receptor tyrosine kinases that together with their ephrin ligands are implicated in various cellular processes, including cell migration, axonal pathfinding, tumorigenesis and boundary formation [1]. In *Drosophila*, a single Eph receptor and a single ephrin have been identified [51], [52]. Mutants in the *Eph* receptor and *ephrin* genes develop into viable and fertile adults [53]. *Eph* receptor mutants display axon guidance defects in the mushroom body [53].

In vertebrate embryos, Eph receptors and their ligands have previously been shown to mediate cell sorting at compartment boundaries between rhombomeres in the hindbrain [1]. The expression of some Eph receptors, and their ephrin ligands, is specific to odd and even-numbered rhombomeres. EphA4 and specific EphB receptors, for example, are expressed in rhombomeres r3 and r5, while ephrin B ligands are expressed in r2, r4, and r6 [54], [55], [56], [57], [58]. Inhibition of EphA4 receptor activity disrupts rhombomere boundaries and mosaic overexpression of EphA4 receptor or ephrin B results in cell sorting [18], [59]. Recent evidence indicates that signaling between Eph receptor and ephrin restricts cell intermingling by increasing adhesion within rhombomeres while decreasing adhesion across rhombomere boundaries (reviewed in [1]).

Our observation that mosaic knock-down of the *Eph* gene results in smooth-edged clones throughout the wing imaginal disc is consistent with the uniform expression of the *Eph* gene that we observed by RNA in situ hybridization (Fig. S1B, C). It will be interesting to determine the expression pattern of the *ephrin* gene and the spatial activation of Eph-ephrin signaling in the wing imaginal disc.

Individual clones of cells expressing double-stranded RNA targeting the *Eph* gene did not alter the shape of the AP boundary. Only when two clones from neighboring compartments abut at the AP boundary, the shape of the AP boundary is distorted. This observation indicates that differences in Eph receptor activity might be important to maintain a straight AP boundary. Further analysis will be required to test the role of Ephrin in the formation of the AP boundary and to reveal the mechanisms by which the Eph receptor influences the shape of the AP boundary. Our screen identified a role for the Eph receptor in maintaining the shape of the AP boundary in *Drosophila* wing imaginal discs. The maintenance of compartment boundaries may therefore be a more common role of Eph receptors not restricted to vertebrates.

## Materials and Methods

### Fly stocks

The genotype of the tester stock used in the screening was *y w hs-flp*; *en*-Venus; *Act5C>CD2>GAL4*, *UAS-DsRed*.

The tester stock was crossed to RNAi lines and offspring was raised at 25°C for 3 days and then subjected to heat shock at 37°C for 40 min to induce somatic clones. Larvae were then kept at 25°C for 3 days before dissection.

RNAi lines were obtained from the Vienna Drosophila RNAi Center (VDRC), the National Institute of Genetics (NIG-FLY), and the Transgenic RNAi Project (TRiP) at Harvard Medical School. Additional stocks used were *amos^1^*, a null allele of *amos* [60], *Sce^1^*, a null allele of *Sce* [61], *en*-Venus [62], and *ap-lacZ* [63].

### Sample preparation

Typically 8 to 10 larvae from each cross were hand dissected to obtain wing imaginal discs. Dissected discs were fixed in PBS with 4% formaldehyde for 40 minutes at room temperature. Fixed samples were washed with PBT (PBS with 0.1% triton) three times and mounted on a glass slide. Phenotypes were evaluated under a standard upright fluorescent microscope (Zeiss).

### Imaging

Images were taken with a Zeiss LSM510 confocal scanning microscope with a 40x NA 1.3 oil objective or a Leica SP5 inverted confocal scanning microscope with a 40x NA 1.2–0.75 oil objective.

### Data analysis

Data handling was carried out in the software for statistical computing and graphics, R 2.15.2. CG numbers for screened RNAi lines (2,153 and 948 lines for VDRC and NIG-FLY stock centers, respectively) were obtained from datasheets provided by the stock centers. Obtained CG numbers (2,011 and 925 for VDRC and NIG-FLY, respectively) were subjected to the ID Converter at the FlyBase web page to obtain the updated FlyBase IDs (32 and 12 CG numbers hit multiple FlyBase IDs for VDRC and NIG, respectively). Updated FlyBase IDs (2,863 in total) were used for the analysis of molecular classification in the PANTHER database (http://www.pantherdb.org/,thomasa [38]). 2,792 genes were mapped (71 were unmapped). The classification was carried out based on the PANTHER Protein Class ontology (identified protein class IDs are indicated in Figure 3A).

### RNA in situ hybridization

RNA in situ hybridization on *yw* late third instar wing imaginal discs was performed as described previously [64]. Plasmid LD14495 was used as template for the preparation of the RNA probes.

Figures were prepared in ScientiFig [65].

## Supporting Information

Figure S1 ***Eph*** **is not required for the maintenance of the DV boundary and is expressed uniformly in larval wing imaginal discs. A.** A wing imaginal disc displaying clones of cells expressing double-stranded RNA targeting *Eph* using the RNAi lines 4771. The clones of cells are identified by the absence of CD2 staining (red). Cells of the dorsal compartment are labeled by expression of *ap-lacZ* (green). Two clones expressing *Eph^dsRNA^* located in different compartments sharing a common interface along the DV boundary (asterisks) do not locally distort the shape of the DV boundary. **B–C**. Late third instar wing imaginal discs hybridized with a sense (B) or antisense (C) *Eph* RNA probe are shown.
(TIF)

Table S1 **List of RNAi lines and associated phenotypes.**
(XLSX)
