# Supplementary figures and images for: An RNA Interference Screen for Genes Required to Shape the Anteroposterior Compartment Boundary in Drosophila Identifies the Eph Receptor
Source: PLoS One. 2014 Dec 4;9(12):e114340. doi: 10.1371/journal.pone.0114340 (PMC4256218; doi:10.1371/journal.pone.0114340)

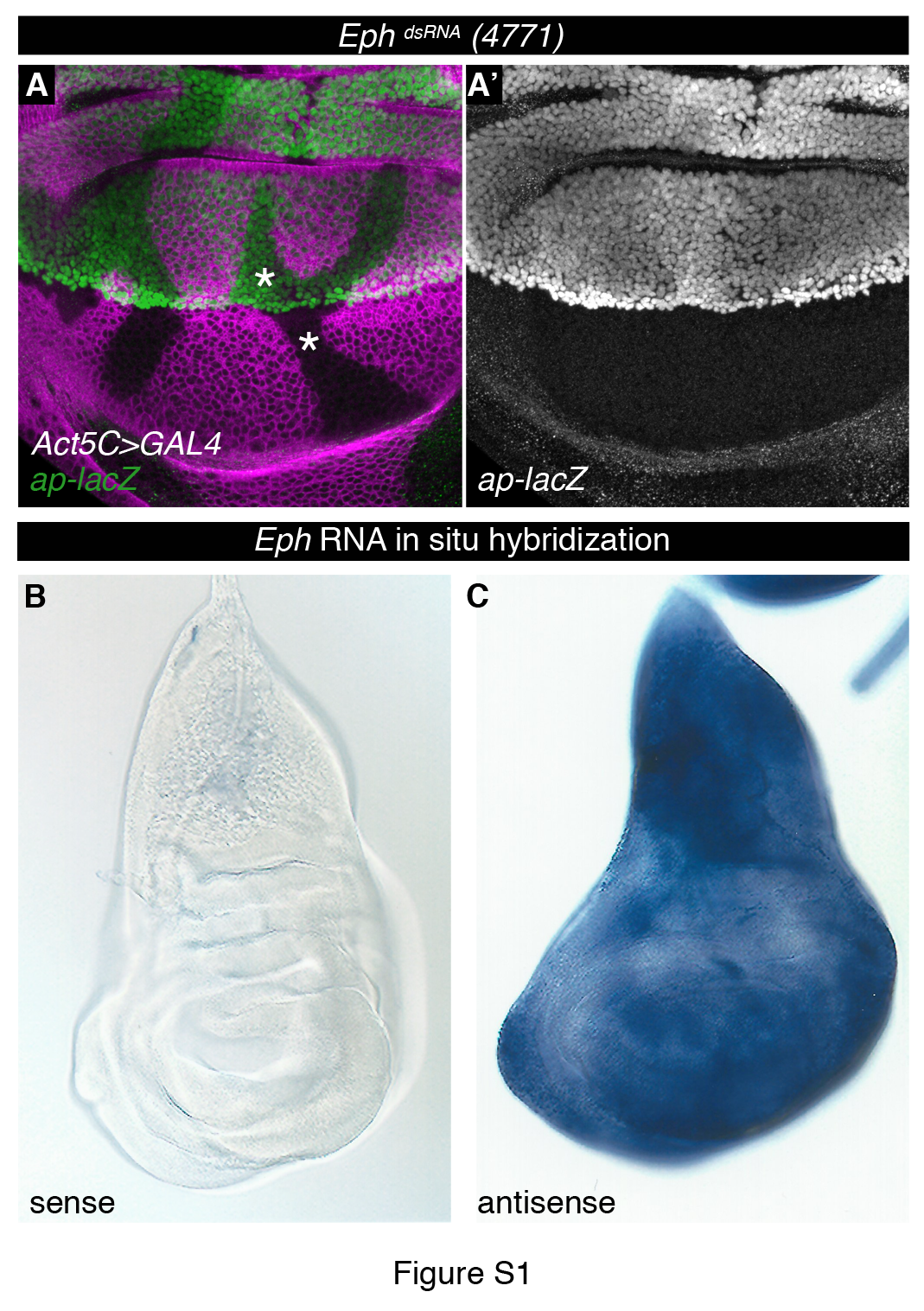

Supplement: Figure S1 — Eph is not required for the maintenance of the DV boundary and is expressed uniformly in larval wing imaginal discs. A. A wing imaginal disc displaying clones of cells expressing double-stranded RNA targeting Eph using the RNAi lines 4771. The clones of cells are identified by the absence of CD2 staining (red). Cells of the dorsal compartment are labeled by expression of ap-lacZ (green). Two clones expressing EphdsRNA located in different compartments sharing a common interface along the DV boundary (asterisks) do not locally distort the shape of the DV boundary. B–C. Late third instar wing imaginal discs hybridized with a sense (B) or antisense (C) Eph RNA probe are shown. (TIF) [file pone.0114340.s001.tif]
